# Regulation of protein O-GlcNAcylation by circadian, metabolic, and cellular signals

**DOI:** 10.1016/j.jbc.2023.105616

**Published:** 2023-12-29

**Authors:** Xianhui Liu, Yao D. Cai, Joanna C. Chiu

**Affiliations:** Department of Entomology and Nematology, College of Agricultural and Environmental Sciences, University of California, Davis, California, USA

**Keywords:** circadian clock, *Drosophila melanogaster*, metabolism, signal transduction, hexosamine biosynthetic pathway, glutamine fructose-6-phosphate aminotransferase, O-GlcNAc processing enzymes, OGT, OGA

## Abstract

O-linked β-*N*-acetylglucosamine (O-GlcNAcylation) is a dynamic post-translational modification that regulates thousands of proteins and almost all cellular processes. Aberrant O-GlcNAcylation has been associated with numerous diseases, including cancer, neurodegenerative diseases, cardiovascular diseases, and type 2 diabetes. O-GlcNAcylation is highly nutrient-sensitive since it is dependent on UDP-GlcNAc, the end product of the hexosamine biosynthetic pathway (HBP). We previously observed daily rhythmicity of protein O-GlcNAcylation in a *Drosophila* model that is sensitive to the timing of food consumption. We showed that the circadian clock is pivotal in regulating daily O-GlcNAcylation rhythms given its control of the feeding-fasting cycle and hence nutrient availability. Interestingly, we reported that the circadian clock also modulates daily O-GlcNAcylation rhythm by regulating molecular mechanisms beyond the regulation of food consumption time. A large body of work now indicates that O-GlcNAcylation is likely a generalized cellular status effector as it responds to various cellular signals and conditions, such as ER stress, apoptosis, and infection. In this review, we summarize the metabolic regulation of protein O-GlcNAcylation through nutrient availability, HBP enzymes, and O-GlcNAc processing enzymes. We discuss the emerging roles of circadian clocks in regulating daily O-GlcNAcylation rhythm. Finally, we provide an overview of other cellular signals or conditions that impact O-GlcNAcylation. Many of these cellular pathways are themselves regulated by the clock and/or metabolism. Our review highlights the importance of maintaining optimal O-GlcNAc rhythm by restricting eating activity to the active period under physiological conditions and provides insights into potential therapeutic targets of O-GlcNAc homeostasis under pathological conditions.

Protein O-linked β-*N*-acetylglucosamine (O-GlcNAcylation) is a unique type of glycosylation, where N-acetylglucosamine (GlcNAc) dynamically cycles on serine and threonine residues of proteins. Since its first discovery in the 1980s in lymphocytes (1), O-GlcNAcylation has been widely found in over 16,000 proteins to date in all domains of life (2). These O-GlcNAcylated proteins are involved in a wide range of fundamental biological processes (3, 4, 5, 6, 7, 8), including transcription, translation, nutrient sensing, immune response, cell signaling, cell cycle, and circadian clocks. Importantly, aberrant O-GlcNAcylation has become the hallmark of many diseases (5, 9, 10, 11, 12), such as cancer, neurodegenerative diseases, cardiovascular diseases, insulin resistance, and type 2 diabetes.

Given the indispensable role of O-GlcNAcylation under both physiological and pathological conditions, it is critical to understand the mechanisms that regulate the cycling of O-GlcNAcylation. O-GlcNAcylation is conventionally considered nutrient-sensitive because it is dependent on the substrate UDP-GlcNAc, the end product of the hexosamine biosynthetic pathway (HBP). HBP is a critical pathway that integrates glucose, amino acid, lipid, and nucleotide metabolism (5). After UDP-GlcNAc is produced, O-GlcNAc transferase (OGT) catalyzes the addition of GlcNAc groups onto proteins, *i.e.* O-GlcNAcylation (13, 14, 15), while O-GlcNAcase (OGA) catalyzes the removal of GlcNAc group (16, 17). Indeed, metabolic input determines substrate availability of O-GlcNAcylation (8, 18, 19, 20, 21). Decades of investigations also uncovered the regulation of O-GlcNAcylation by metabolic input *via* the modulation of HBP enzymes and O-GlcNAc processing enzymes.

Accumulating evidence showed that many other cellular signals and conditions also contribute to the regulation of protein O-GlcNAcylation. Notably, the circadian clock has been shown to shape the daily rhythm of protein O-GlcNAcylation (18). The circadian clock is an endogenous biochemical timer that allows organisms to anticipate predictable environmental changes over the 24-h day-night cycles, such as daily light-dark cycles and temperature cycles (22, 23, 24, 25). In animals, daily oscillations of physiological, metabolic, and behavioral processes are strongly regulated by circadian clocks (22, 23, 24, 25). Therefore, the regulation of protein O-GlcNAcylation by circadian clocks could be imposed at multiple levels. At the behavioral level, the circadian clock controls daily feeding-fasting behavior to rhythmically provide metabolic input for UDP-GlcNAc production and O-GlcNAcylation (18). Indeed, under physiological conditions, the daily feeding-fasting cycle is shown to correlate with daily O-GlcNAcylation rhythm in mouse hearts (26) and *Drosophila* tissues (18). At the cellular level, circadian clocks can orchestrate daily rhythms in mRNA, protein abundance, and/or enzymatic activities of HBP enzymes and O-GlcNAc processing enzymes (18, 27, 28, 29). Finally, a number of other factors also influence O-GlcNAcylation, such as endoplasmic reticulum (ER) stress, oxidative stress, infection, apoptosis, development, and aging, to name a few (30, 31, 32, 33, 34, 35, 36, 37, 38, 39, 40).

In this review, we will provide a broad overview of the metabolic, circadian, and cellular signals that regulate protein O-GlcNAcylation. We will summarize the effect of nutrient availability on HBP and O-GlcNAcylation, highlight the metabolic regulation of protein O-GlcNAcylation through impacting HBP and O-GlcNAc processing enzymes, discuss the evidence on the regulation of daily O-GlcNAcylation rhythms by the circadian clock, and outline other cellular signals that modulate protein O-GlcNAcylation.

## The regulation of protein O-GlcNAcylation by nutrient availability

Depending on the cell type and metabolic status of the cell, 0.003% to 3% of the glucose in cells feeds into the HBP pathway (41, 42, 43). In primary cultured adipocytes, Marshall *et al.* (41) indirectly estimated that 2% to 3% of glucose feeds into the HBP by measuring the relative glucosamine *versus* glucose utilization of the cells. Gibb *et al.* (42) concluded that the HBP in cultured neonatal cardiomyocytes utilizes more glucose than the pentose phosphate pathway by measuring the overall levels of isotope-labeled UDP-HexNAc. Their study suggested that previous studies severely underestimated glucose flux into the HBP. Olson *et al.* (43) directly monitored glucose flux through the HBP using isotope tracking in an isolated working mouse heart and observed that only 0.003% to 0.006% of glucose is metabolized through the HBP. Future investigations are warranted to determine the glucose fluxes into the HBP in different cell types, organs, and species and investigate how glucose fluxes are differentially regulated in different cell types and organs.

**Figure 1:**
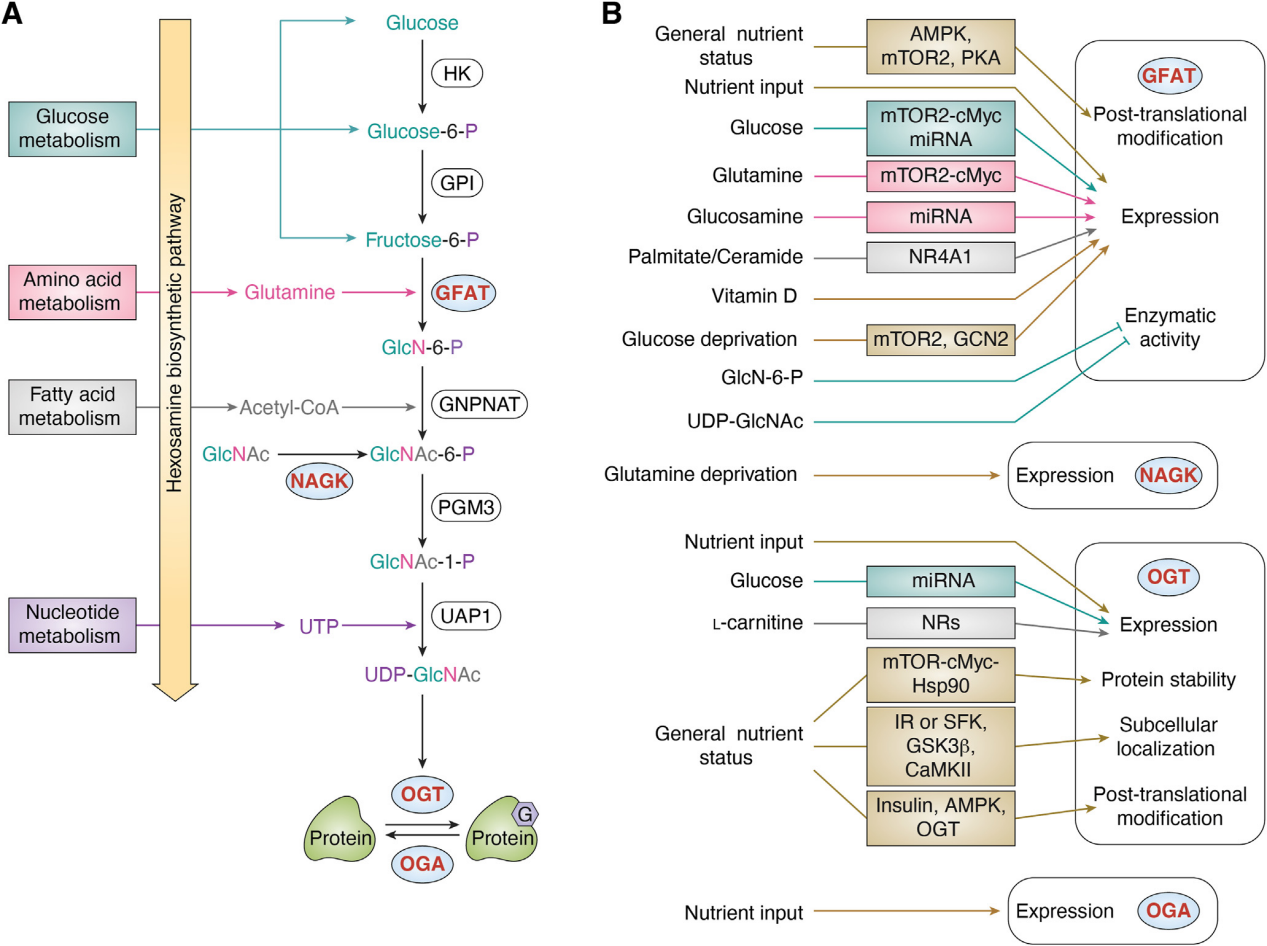
**Metabolic regulation of protein O-GlcNAcylation.***A*, nutrient input from feeding activity or cell culture media provides essential metabolites for hexosamine biosynthetic pathway (HBP). The end product of HBP, UDP-GlcNAc, is the substrate for protein O-GlcNAcylation. The key enzymes that are under additional metabolic regulation are highlighted in blue circle. *B*, nutrient level regulates the activities of glutamine:fructose-6-phosphate amidotransferase (GFAT) (13, 44, 54, 60, 61, 62, 63, 64, 66, 67, 68, 69, 70, 71, 72, 73, 76, 77), N-acetylglucosamine kinase (NAGK) (78), O-GlcNAc transferase (OGT) (13, 14, 55, 79, 80, 81, 82, 83, 84, 85, 86, 87, 88, 89, 90, 91) and O-GlcNAcase (OGA) (13). These enzymes represent hubs for metabolic regulation of O-GlcNAcylation. The expression denotes mRNA and/or protein expression of indicated enzymes. Functional groups of each metabolite are color-coded to highlight the processes that contribute to their metabolism. Other metabolites or nutrient status that contribute to protein O-GlcNAcylation are illustrated in *brown*. O-GlcNAc is depicted as G on protein. AMPK, AMP-activated protein kinase; CaMKII, calcium–calmodulin (CaM)-dependent protein kinase II; GCN2, general control nonderepressible 2; GFAT, glutamine–fructose-6-phosphate aminotransferase; GlcN-6-P, glucosamine-6-phosphate; GlcNAc, N-acetylglucosamine; GlcNAc-1-P, N acetylglucosamine-1-phosphate; GlcNAc-6-P, N-acetylglucosamine-6-phosphate; GNPNAT, glucosamine-phosphate N-acetyltransferase; GPI, phosphoglucose isomerase; GSK3β, glycogen synthase kinase-3 β; HK, Hexokinase; Hsp90, Heat shock protein 90; IR, insulin receptor; miRNA, microRNA; mTOR, mechanistic target of rapamycin; NR, nuclear receptor; NR4A1, nuclear subfamily four group A member one; OGA, O-GlcNAcase; OGT, O-GlcNAc transferase; PGM3, phosphoacetylglucosamine mutase; PKA, protein kinase A; PTM, post-translational modification; SFK, Src family kinases, UAP1, UDP-N-acetyl glucosamine pyrophosphorylase one; UDP-GlcNAc, uridine diphosphate N-acetylglucosamine; UTP, uridine triphosphate.

The HBP then converts metabolites from amino acid, lipid, and nucleotide metabolism to produce UDP-GlcNAc (Fig. 1*A*). Since the activity of OGT is highly responsive to UDP-GlcNAc concentration with multiple K_m_ values (44), the effect of nutrient availability on UDP-GlcNAc level has been of great interest. Generally, UDP-GlcNAc levels in many tissue culture experiments have been observed to change in accordance with the levels of various nutrients, including glucose, glutamine, glucosamine, acetylglucosamine, uridine, palmitate, and stearate (19, 20, 21, 45, 46, 47, 48, 49) (Fig. 1*B*). Moreover, the correlation between nutrient availability and UDP-GlcNAc has also been investigated in animals. The UDP-GlcNAc level has been shown to increase in rat skeletal muscles after infusion of lipid emulsion, uridine, or GlcN for 7 h (50). In *Drosophila*, the daily UDP-GlcNAc level correlates with feeding rhythm under *ad libitum* conditions (18). This correlation has been confirmed by time-restricted feeding paradigms. When the fly feeding activity was restricted to different 6-h windows within a day-night cycle, the timing of the peak of the daily UDP-GlcNAc rhythm shifted according to the timing of food consumption.

Experiments to determine whether protein O-GlcNAcylation level corresponds to nutrient availability have produced mixed results so far. In some cell lines or *ex vivo* tissues, O-GlcNAcylation levels increase as the level of glucose, glucosamine, and glutamine increases (51, 52, 53, 54) and vice versa (19, 55). Similarly, under pathological conditions, excessive glucose availability during hyperglycemia results in higher O-GlcNAcylation levels (11, 56, 57, 58). *In vivo* studies in mouse heart and fly tissues also reported that daily O-GlcNAcylation rhythm is correlated with feeding-fasting cycles and nutrient availability (18, 26). Nevertheless, glucose starvation has been shown to increase O-GlcNAcylation in several studies (19, 59, 60). In addition, when flies are fed at unnatural feeding time (*i.e.* when flies are awake but normally fasting), O-GlcNAcylation rhythm does not shift its peak time to coincide with the shifted time of food consumption, but rather feeding at unnatural time results in dampened O-GlcNAcylation rhythm (18). On one hand, glucose starvation or mistimed eating could modulate OGT, OGA, and glutamine fructose-6-phosphate aminotransferase (GFAT), the rate-limiting enzyme of HBP (See sections below) (19, 59, 60), thus creating more complex scenarios rather strict correlation between nutrient availability and O-GlcNAcylation level. Indeed, mistimed eating alters *ogt* and *oga* mRNA levels, as well as GFAT enzymatic activity (18). On the other hand, it is very likely that the response of protein O-GlcNAcylation to glucose starvation or mistimed eating could be cell type or tissue-specific, as Pham *et al.* (55) demonstrated that nutrient deprivation differentially influences protein O-GlcNAcylation in a number of cell lines.

In conclusion, despite that nutrient availability mostly determines UDP-GlcNAc production in cell lines and tissues, cellular O-GlcNAcylation level does not always correspond to nutrient availability. This suggests that additional cellular factors regulate protein O-GlcNAcylation. Future investigations into conservation and differences in the regulation of UDP-GlcNAc production and O-GlcNAcylation by nutrient availability in different organs and species, especially diurnal *versus* nocturnal animals, will certainly provide new insights into the regulation of protein O-GlcNAcylation by nutrient availability.

## Metabolic regulation of protein O-GlcNAcylation through regulation of HBP enzymes

In addition to nutrient availability, the activity of HBP enzymes also plays critical roles in UDP-GlcNAc production. Four key enzymes are involved in HBP: GFAT, glucosamine-phosphate N-acetyltransferase (GNPNAT), GlcNAc phosphomutase (PGM3/AGM1), and UDP-N-acetylglucosamine pyrophosphorylase (UAP1/AGX1) (Fig. 1*A*).

As the rate-limiting enzyme of HBP (61), GFAT is the most studied to date. In animals, two GFAT paralogs, GFAT1 and GFAT2, share 75% to 80% amino acid sequence. They are functionally equivalent but distributed in different tissues or cell types (62, 63, 64). At the mRNA level, *gfat* is responsive to metabolic signals (Fig. 1*B*). Many macronutrients stimulate *gfat* mRNA expression, including glucose (65, 66, 67), glutamine (67), glucosamine (65), palmitate (49, 68), stearate (49), and ceramide (68) (Fig. 1*B*). The effect of glucose and glutamine on *gfat1* expression is mediated by mTOR2-cMYC axis (67), while nuclear receptor subfamily four group A member 1 (NR4A1) transcription factor can respond to palmitate and ceramide levels to increase *gfat2* mRNA (68). Moreover, glucose and glucosamine can upregulate *gfat* mRNA levels by inhibiting microRNA (miR)-27b-3p (65) (Fig. 1*B*). In addition to evidence from cell culture experiments described above, we showed that *gfat2* mRNA in fly tissues, but not *gfat1* mRNA, is strongly responsive to nutrient input *via* feeding activity (18). On the contrary, nutrient deprivation also increases *gfat1* mRNA expression. This could be mediated by mTOR2 (69) and/or general control nonderepressible 2 (GCN2)-activating transcription factor 4 (ATF4) pathways (59) (Fig. 1*B*). Although these observations are counterintuitive considering the nutrient sensitivity of HBP, the increased *gfat1* expression is speculated to have protective roles upon moderate nutrient deprivation (59, 69). In addition to macronutrients, vitamin D can inhibit *gfat* expression in rat hearts through an unknown mechanism (70) (Fig. 1*B*).

At the posttranslational level, GFAT is phosphorylated by nutrient-sensing kinases, including AMP-activated protein kinase (AMPK), mTOR complex 2 (mTORC2), and protein kinase A (PKA) (Fig. 1*B*). AMPK, an energy sensor, phosphorylates GFAT1 at serine 243 (S243) (71, 72). mTORC2 also phosphorylates S243 in response to glucose and glutamine concentration in cell media (73). Furthermore, nucleoside diphosphate kinase B (NDPKB) counteracts S243 phosphorylation through an unknown mechanism (74). Despite that the kinases involved are characterized, how S243 phosphorylation modulates GFAT activity remains controversial. Moloughney *et al.* (73) reported that S243 phosphorylation increases GFAT1 activity, while Eguchi *et al.* (71) showed that S243 phosphorylation downregulates GFAT1 activity. This could be due to different cell lines utilized in these studies (mouse embryonic fibroblasts and HeLa cells in (73), Chinese hamster ovary cells overexpressing insulin receptors in (71)). Finally, under fasting or starvation conditions, GFAT exhibits lower activity (75, 76), which could be mediated by PKA (75) (Fig. 1*B*). PKA-directed S205 phosphorylation reduces GFAT1 activity (75, 77). The function of PKA-dependent GFAT1(S235) phosphorylation remains unknown (75). On the contrary, PKA is also shown to increase GFAT activity in two other studies (78, 79). Given that PKA also phosphorylates GFAT2(S202) and increases GFAT2 activity by 2.2-fold (80), increased GFAT2 activity may counteract the PKA-dependent reduction of GFAT1 activity (79). Therefore, the effect of PKA on GFAT activity could exhibit cell type specificity depending on relative expression levels of GFAT1 and GFAT2.

Finally, HBP metabolites can feedback to regulate GFAT activity. In the early 2000s, *in vitro* studies showed that glucosamine-6-phosphate (GlcN-6-P), the immediate product of the GFAT-catalyzed reaction, and UDP-GlcNAc both inhibit GFAT activity (78, 81) (Fig. 1*B*). Follow-up structural biology analysis showed that UDP-GlcNAc interacts with an interdomain linker to allosterically downregulate GFAT activity (82).

In summary, significant progress has been made to uncover the metabolic regulation of *gfat* mRNA and GFAT protein expression as well as its enzymatic activity. In comparison, studies that examined the metabolic regulation of other HBP enzymes are limited. One recent study revealed that glutamine deprivation increases UDP-GlcNAc levels *via* N-acetylglucosamine kinase (NAGK)-dependent salvage of GlcNAc, another precursor of UDP-GlcNAc (83). Future investigations of other HBP enzymes will be important to provide a comprehensive understanding of the metabolic regulation of protein O-GlcNAcylation.

## Metabolic regulation of protein O-GlcNAcylation through regulation of O-GlcNAc processing enzymes

OGT utilizes UDP-GlcNAc to modify proteins, while OGA reverses this modification. In this section, we discuss the mechanistic regulation of OGT and OGA by nutrient availability and nutrient sensing pathways. Nutrient availability and nutrient sensing pathways can regulate OGT protein expression (Fig. 1*B*). Feeding activity and hence nutrient availability have been shown to regulate daily OGT expression rhythm in *Drosophila* animals (18). When flies are subjected to time-restricted feeding (TRF) at different time windows, robust daily oscillation of OGT protein is observed. The peak time of OGT protein rhythm coincides well with the timing of food consumption (18). A similar phenomenon has been observed at the *ogt* mRNA level under TRF conditions, except for two minor differences: (a) the amplitude of *ogt* mRNA rhythm remains the same when the timing of food consumption is altered and (b) the peak time of *ogt* mRNA is delayed by a few hours after feeding time (18).

A number of other studies investigated the effect of glucose on OGT expression, producing somewhat contradictory conclusions. Under high glucose conditions, glucose can promote *ogt* mRNA and subsequently protein levels through inhibiting miR-200a/b (84) (Fig. 1*B*). Interestingly, *ogt* mRNA and OGT protein levels have also been shown to be elevated under glucose deprivation (19, 60), although the mechanism is unknown. More recently, L-carnitine has been shown to regulate *ogt* mRNA expression through the activity of nuclear receptors (85) (Fig. 1*B*). Finally, it has been reported that mTOR nutrient-sensing pathway can promote OGT protein levels *via* the cMYC-Hsp90 axis (86, 87, 88) (Fig. 1*B*).

Regulation of mRNA and protein levels is not the only mechanism by which O-GlcNAc processing enzymes are regulated by metabolic signals. OGT subcellular localization is modulated by nutrient-sensing pathways to achieve temporal O-GlcNAcylation of proteins in different cellular compartments. OGT contains a nuclear localization sequence (NLS) (residue 451–453). Upon O-GlcNAcylation of OGT(S389), importin α5 recognizes the NLS and imports OGT into the nucleus (89) (Fig. 1*B*). Given that O-GlcNAcylation is responsive to metabolic signals as described in previous sections, OGT(S389) O-GlcNAcylation could be one of the mechanisms by which metabolic signals regulate OGT. In addition, in response to the glucose level in cell media, AMPK also modulates the nuclear translocation of OGT by OGT(T444) phosphorylation, probably through importin α5-dependent mechanisms (90) (Fig. 1*B*). Finally, the insulin signaling pathway has been shown to recruit OGT to the cytoplasm (91, 92). Upon insulin stimulation, PI(3,4,5)P3 binds the PIP-binding domain of OGT and promotes cytoplasmic localization (92) (Fig. 1*B*).

Nutrient-sensitive OGT phosphorylation can directly control its enzymatic activity. Insulin signaling promotes phosphorylation of yet uncharacterized tyrosine residue(s) on OGT, possibly by insulin receptor and/or Src protein-tyrosine kinase (91) (Fig. 1*B*). Downstream of insulin signaling, glycogen synthase kinase 3β (GSK3β) phosphorylates OGT(S3/4) (93) (Fig. 1*B*). Wang *et al.* (94) further showed that inhibiting GSK3β alters OGT substrate selectivity, adding an additional layer of OGT regulation by insulin signaling. During nutrient deprivation, calcium-calmodulin-dependent protein kinase II (CaMKII) phosphorylates OGT(S20) and increases OGT activity (95, 96) (Fig. 1*B*).

Similar to OGT, OGA protein levels are highly dependent on the timing of food consumption in *Drosophila* animals (18). At the transcript level, *oga* mRNA is rhythmic when flies feed at natural feeding time but not at unnatural feeding time when they are normally fasting (18), indicating *oga*/OGT is regulated by both nutrient input *via* feeding and circadian clocks (see next section). At present, the molecular mechanisms by which *oga* mRNA and OGA protein are regulated by nutrient availability and/or nutrient sensing pathways are largely unknown. One potential mechanism is that nutrient-sensitive O-GlcNAcylation can modify OGA (97). However the functional outcome of OGA O-GlcNAcylation remains a gap in knowledge. In conclusion, OGT can be regulated by metabolic signals in multiple ways, and more studies are warranted to investigate the metabolic regulation of OGA.

## Regulation of daily protein O-GlcNAcylation rhythm by the circadian clock

**Figure 2:**
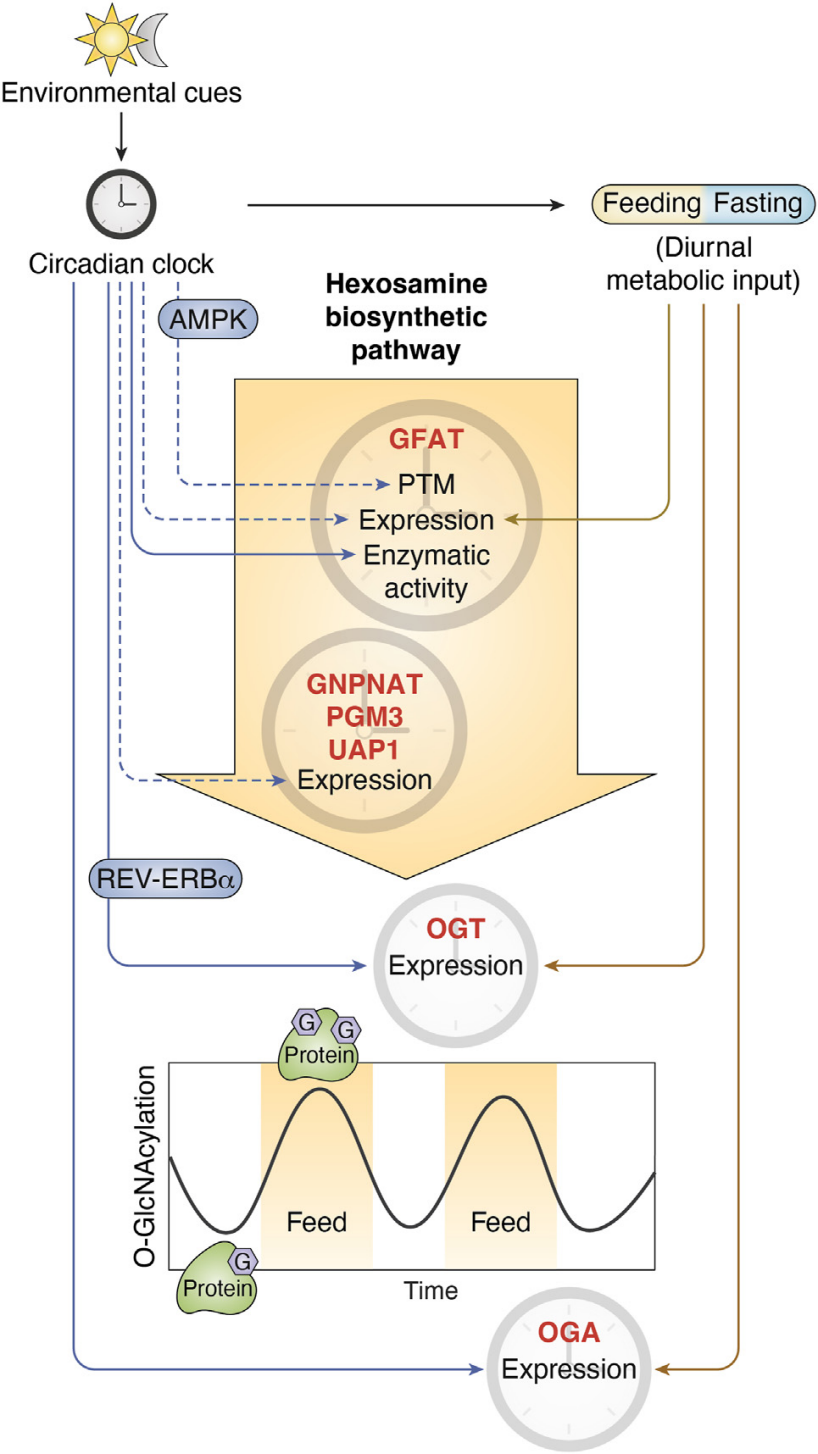
**The circadian clock and metabolic signals from the feeding-fasting cycle shape the daily oscillation of protein O-GlcNAcylation.** The circadian clock receives environmental signals, such as *light-dark cycles*, and regulates the daily feeding-fasting cycle. Feeding-fasting cycle rhythmically provides nutrient input for the hexosamine biosynthetic pathway (HBP) and contributes to the daily rhythmic production of UDP-GlcNAc and protein O-GlcNAcylation rhythms (18). The circadian clock and feeding-fasting cycle also regulate protein O-GlcNAcylation rhythm by modulating the activity of HBP enzymes (13, 22, 23, 24, 93, 94) and O-GlcNAc processing enzymes (13, 23, 24, 88, 93, 95, 96, 97, 98, 99, 100, 101, 102, 103, 104, 105). Enzymes under daily rhythmic regulation are annotated with clock cartoons. The expression denotes mRNA and/or protein expression of indicated enzymes. *Solid arrows* indicate evidence from targeted studies of individual proteins, while *dashed arrows* indicate evidence gathered from omics studies. Line color differentiates clock-driven regulation (*blue*) and feeding-driven regulation (*brown*). AMPK and REV-ERBα are potential molecular mediators of clock-driven regulation on GFAT (71, 72, 98) and OGT (99), respectively. AMPK, AMP-activated protein kinase; GFAT, glutamine–fructose-6-phosphate aminotransferase; GNPNAT, glucosamine-phosphate N-acetyltransferase; OGA, O-GlcNAcase; OGT, O-GlcNAc transferase; PGM3, phosphoacetylglucosamine mutase; UAP1, UDP-N-acetyl glucosamine pyrophosphorylase one.

Given the nutrient sensitivity of protein O-GlcNAcylation discussed in the previous sections, it is perhaps not surprising that protein O-GlcNAcylation oscillates over the 24-h day–night cycle due to rhythmic feeding-fasting behaviors in animals. Indeed, both mice and flies display daily protein O-GlcNAcylation rhythms, primarily peaking during their feeding windows (18, 26) (Fig. 2). Interestingly, several lines of evidence suggest circadian clocks regulate protein O-GlcNAcylation through other mechanisms in addition to the regulation of rhythmic feeding-fasting behavior. First, when flies are forced to feed at unnatural feeding time, protein O-GlcNAcylation rhythm dampens significantly. This indicates circadian clocks may provide buffering mechanisms that prevent protein O-GlcNAcylation from peaking at nonoptimal time. Second, in flies with defective circadian clocks, protein O-GlcNAcylation rhythm also dampens significantly and the levels remain constant throughout a 24-h period (18). This is not purely due to disrupted feeding-fasting activities, given that restricting the feeding time of clock mutant flies at natural feeding time does not rescue O-GlcNAcylation rhythms. We devote this section to reviewing the evidence on the regulation of daily O-GlcNAcylation rhythm by the circadian clock.

In addition to rhythmic UDP-GlcNAc production driven by clock-controlled feeding activity (18), the activity of HBP enzymes could also be under clock control. Based on publicly available circadian transcriptomic datasets in flies and mice, we found that the transcripts encoding all four HBP enzymes oscillate over a day-night cycle in at least one study (Fig. 2 and Table 1). At the protein level, published proteomic datasets suggest that GFAT, GNPNAT, and UAP1 proteins exhibit daily oscillation (Fig. 2 and Table 1). As for the enzymatic activity of HBP enzymes, our recent study reported daily oscillation of GFAT enzymatic activity in wild type (WT) fly tissues, but this rhythmicity is diminished in arrhythmic clock mutant flies (*per*^*0*^*; period* null mutant fly) (18). Interestingly, restricting food consumption of clock mutant flies at natural feeding time of WT flies failed to rescue rhythmic GFAT enzymatic activity. This suggests that a functional circadian clock is essential to maintain GFAT activity rhythm beyond the regulation of feeding-fasting cycles. Given our observation that the peak of daily GFAT activity rhythm is always fixed right after the natural feeding time window in WT flies, even when flies were fed at unnatural feeding time (18), we concluded that circadian clocks impose a stronger regulation on GFAT activity rhythm as compared to nutrient input (Fig. 2). Clock-controlled GFAT regulation likely happens at posttranscriptional and/or posttranslational levels (18). At present, it remains unknown whether the clock-controlled daily feeding-fasting cycle shapes daily GFAT activity rhythm through metabolic regulations (See previous sections). Intriguingly, phosphoproteomics in mouse liver tissue detected rhythmic GFAT1(S259) phosphorylation over a 24-h period and occupancy of this phosphorylation peaks right after mouse feeding phase (98) (Fig. 2). Mouse GFAT1(S259) is equivalent to human GFAT1(S243), the AMPK-directed phosphorylation with still debatable effects on GFAT1 activity as discussed above. Finally, whether rhythmic GFAT1(S259) phosphorylation leads to rhythmic GFAT activity remains to be investigated.

**Table 1:** Circadian regulation of hexosamine biosynthetic pathway and O-GlcNAc cycling enzymes in fly or mouse tissues based on published data

Enzymes	Mouse			Fly		Reference
	Transcripts	Protein	Phospho-peptide	Transcripts	Protein	
GFAT/GFPT	Cycling in liver, SCN, BAT, kidney, and aorta	Cycling in macrophages	Cycling in liver	Cycling in head and body	Non-cycling in head	(18, 28, 29, 98, 173, 197, 198, 199, 200, 201, 202)
GNPNAT	Cycling in liver	Cycling in macrophages	N/A	Cycling in head	Cycling in head	(27, 29, 197, 202, 203, 204)
PGM3	Cycling in liver, urinary bladder, lung, and AG	Cycling in macrophages	N/A	Non-cycling in head	Cycling in head	(27, 28, 29, 197, 199, 202, 205, 206, 207, 208, 209)
UAP1	Cycling in kidney, lung and WAT	Cycling in liver and macrophages	N/A	Non-cycling in head	Cycling in head	(27, 28, 29, 197, 198, 199, 202, 206, 207, 209, 210)
OGT	Cycling in liver, SCN and DC	Cycling in macrophages	N/A	Cycling in body	Cycling in body	(18, 27, 28, 29, 93, 173, 197, 199, 202, 206, 210, 211, 212, 213)
OGA	Cycling in liver	Cycling in liver and macrophages	N/A	Cycling in head and body	Cycling in head and body	(18, 27, 28, 29, 198, 202, 203, 205, 211, 214, 215, 216)

Abbreviations: AG, adrenal gland; BAT, brown adipose tissue; DC, distal colon; GFAT/GFPT, Glutamine--fructose-6-phosphate aminotransferase; GNPNAT, Glucosamine-phosphate N-acetyltransferase; N/A, Undetected; OGA, O-GlcNAcase; OGT, O-GlcNAc transferase; PGM3, Phosphoacetylglucosamine mutase; SCN, suprachiasmatic nucleus; UAP1, UDP-N-Acetyl glucosamine pyrophosphorylase one; WAT, white adipose tissue.

Other than HBP enzymes, OGT and OGA are also under circadian clock regulation. Combing through published circadian transcriptomic and proteomic datasets, we found that both transcript and protein levels of *oga* and *ogt* are rhythmic in mouse and fly tissues (Fig. 2 and Table 1). In our TRF experiments, the respective mRNA and protein of OGT and OGA are all rhythmic in fly body tissues (18) (Fig. 2). More importantly, the rhythmicity of OGT and OGA protein expression is diminished in arrhythmic clock mutant flies (*per*^*0*^) even under natural feeding time (18), suggesting the requirement of an intact circadian clock to properly regulate the rhythm of OGT and OGA proteins. Finally, in human hepatoma HepG2 cells, REV-ERBα, a core component of the molecular clock, can bind and stabilize OGT (99) (Fig. 2). As REV-ERBα translocates between nucleus and cytoplasm over a day-night cycle, OGT stability in different cellular compartments can also oscillate (99).

In summary, the expression of HBP enzymes and O-GlcNAc processing enzymes are highly regulated by circadian clocks. The phosphorylation and enzymatic activity of GFAT are also rhythmic over a 24-h period. Therefore, under physiological conditions, it is essential to maintain a proper feeding-fasting cycle as established by circadian clocks to maintain an O-GlcNAcylation homeostasis. The regulation of daily O-GlcNAcylation rhythm by the circadian clock is likely to be more extensive than discussed here. Currently, it is unknown whether other HBP enzymes and O-GlcNAc processing enzymes are also rhythmically phosphorylated or rhythmically modified by other posttranslational modifications (PTMs) and whether the activities of other HBP enzymes and O-GlcNAc processing enzymes oscillate over 24-h day-night cycles.

## A wide range of cellular signals regulate protein O-GlcNAcylation

**Figure 3:**
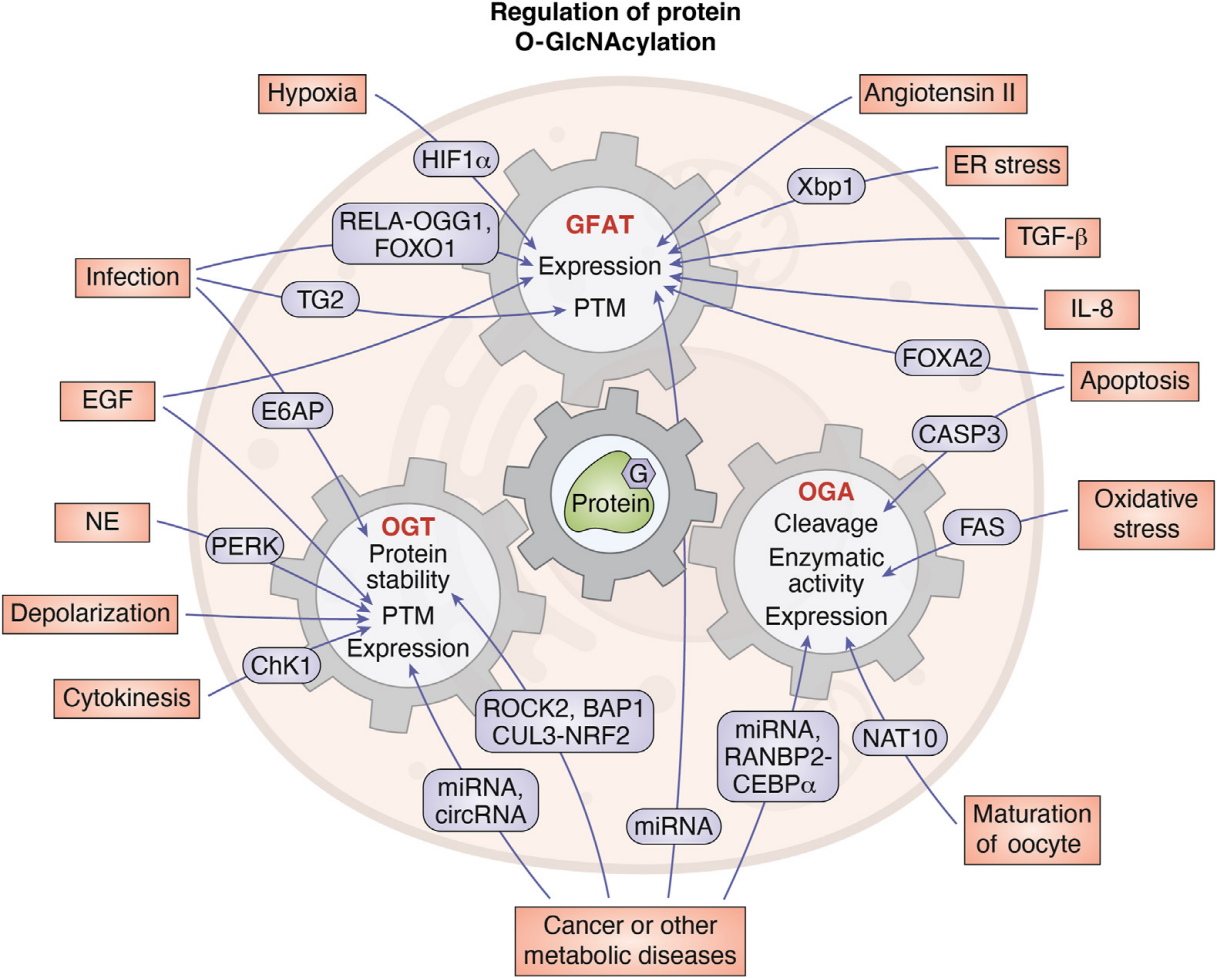
**The regulation of protein O-GlcNAcylation by cellular signals and conditions.** Protein O-GlcNAcylation can respond to a wide range of cellular signals and conditions, mediated by GFAT (61, 106, 107, 108, 109, 110, 111, 112, 113, 114, 115, 116, 143), OGT (117, 118, 119, 120, 121, 122, 123, 126, 127, 128, 129, 130, 131, 132, 133, 134, 135, 136, 139, 140, 141, 142, 146, 147) and OGA (117, 124, 125, 137, 138, 139, 144). The expression denotes mRNA and/or protein expression of indicated enzymes. Known regulators involved are shown and discussed in the text. BAP1, BRCA1 associated protein-1; CASP3, caspase-3; CEBPα, CCAAT/enhancer-binding protein α; ChK1, checkpoint kinase one; circRNA, circular RNA; CUL3, cullin three; E6AP, ubiquitin ligase E6AP; EGF, epidermal growth factor; FAS, fatty acid synthase; FOXA2, forkhead box A2; FOXO1, forkhead box protein O1; HIF1α, hypoxia-inducible Factor; IL-8, interleukin-8; miRNA, microRNA; NAT10, N-acetyltransferase 10; NE, Norepinephrine; NRF2, nuclear factor E2-related factor-2; OGG1, 8-oxoguanine DNA glycosylase one; PERK, protein kinase R-like ER kinase; RANBP2, RAN-binding protein two; RELA, nuclear factor-κB p65; ROCK2, Rho-associated coiled-coil forming protein kinase two; TG2, transglutaminase two; TGF-β, transforming growth factor β; Xbp1, spliced X-box binding protein one.

In addition to metabolic and environmental signals communicated through the circadian clock, protein O-GlcNAcylation is also responsive to various other cellular signals, including ER stress, oxidative stress, infection, apoptosis, and cell division (31, 32, 33, 35, 36, 38, 39) (Fig. 3). Significantly, elevated O-GlcNAcylation under many stress conditions facilitates cell survival (31, 32, 36, 39). Cellular signals are known to regulate protein O-GlcNAcylation by modulating the activity of HBP enzymes, specifically GFAT, and O-GlcNAc processing enzymes. In this section, we will discuss the cellular signals and relevant mechanisms that target GFAT, OGT, and OGA to regulate protein O-GlcNAcylation. The examples provided in this section are mostly from mammalian cell culture studies.

Multiple humoral signals, environmental stimuli, and stress conditions influence the expression and PTM of GFAT. Angiotensin II (100), epidermal growth factor (EGF) (66), transforming growth factor β (TGF-β) (101), and interleukin-8 (IL-8) (102) can promote GFAT protein expression *via* yet uncharacterized mechanisms (Fig. 3). Other cellular signals can modify transcription factors that are responsible for *gfat* mRNA expression (103, 104, 105, 106, 107, 108, 109) (Fig. 3). For example, hypoxia can stimulate *gfat1* expression through hypoxia inducible factor 1α (HIF1α) (104). Forkhead box A2 (FOXA2) can up-regulate *gfat1* expression during apoptosis (106). Unfolded protein response can trigger the expression of multiple transcripts that encode HBP enzymes through the activity of spliced X-box binding protein 1 (Xbp1s) (103). Finally, infection increases GFAT enzymatic activity by transglutaminase 2 (TG2)-dependent PTM on GFAT (110) (Fig. 3).

OGT protein and enzymatic activity also respond to various stimuli and stress conditions. Oxidative stress (111), virus infection (112, 113), and cytokinesis (114) have been shown to regulate OGT protein levels (Fig. 3). The E6 protein from human papillomavirus can interact with E6AP E3 ligase to enhance OGT ubiquitination and promote OGT degradation (113) (Fig. 3). During cytokinesis, checkpoint kinase 1 (CHK1) phosphorylates OGT(S20), which stabilizes OGT by decreasing OGT ubiquitination (114) (Fig. 3). Other OGT phosphorylation events have also been reported to regulate OGT activity in response to cellular signals. EGF signaling stimulates OGT(Y976) phosphorylation and alters its substrate selectivity (115) (Fig. 3). Norepinephrine (NE) can promote OGT phosphorylation by protein kinase R-like ER kinase (PERK) and increase OGT activity on casein kinase 2α (CK2α) (116) (Fig. 3). Depolarization of neuronal cells can stimulate OGT activity through CaMKIV-dependent phosphorylation (117) (Fig. 3).

Similar to OGT, OGA is also regulated by a range of cellular conditions. OGA expression and activity have been shown to be responsive to apoptosis (118), oxidative stress (111), and maturation of oocytes (119) (Fig. 3). Apoptosis induces the cleavage of OGA protein by caspase-3 (CASP3), yet interestingly no effect on OGA activity was observed (118) (Fig. 3). Under oxidative stress, OGA activity is diminished by interacting with fatty acid synthase (FAS) (111) (Fig. 3). During *in vitro* maturation of mouse oocytes, N-acetyltransferase 10 (NAT10) increases OGA protein levels by stabilizing *oga* mRNA *via* mRNA N^4^-acetylcytidine (ac4C) (119) (Fig. 3).

In addition to cellular signals and conditions described above, GFAT, OGT and OGA are also altered under pathophysiological conditions (120, 121, 122, 123, 124, 125, 126, 127, 128, 129, 130, 131, 132, 133, 134, 135, 136, 137, 138, 139, 140, 141) (Fig. 3). The misregulation of O-GlcNAcylation has been of great interest in the study of cancers. For example, several microRNAs (123, 124, 125, 126, 127, 128, 129, 130, 135, 140, 141) and a circular RNA (122) are found to negatively regulate OGT activity, while Rho-associated coiled-coil forming protein kinase 2 (ROCK2) (120) and BRCA1 associated protein-1 (BAP1) (121) inhibit OGT ubiquitination and stabilize OGT (Fig. 3). OGA expression could be downregulated by RAN-binding protein 2 (RANBP2), a SUMO E3 ligase, which cause degradation of CCAAT/enhancer-binding protein α (CEBPα), a transcription factor of OGA (131) (Fig. 3).

In summary, O-GlcNAcylation is responsive to a wide range of cellular signals and/or conditions, suggesting the role of O-GlcNAcylation as a more generalized cellular status effector. Interestingly, cellular signals and/or conditions mentioned in this section also respond to metabolic and circadian signals, forming an intertwined network of regulation on O-GlcNAcylation (Table 2). Given the ubiquitous function of O-GlcNAcylation in biological processes and the importance of maintaining O-GlcNAcylation homeostasis, it is worth noting that OGT and OGA levels generally correlate with each other in many different cell types or conditions (18, 60, 132, 133, 142, 143). Also, general cellular O-GlcNAcylation status can feedback to regulate OGT and/or OGA expression (134, 144, 145). The detailed molecular mechanism that maintains cellular O-GlcNAcylation homeostasis has been summarized in other review articles (5, 6).

**Table 2:** Some cellular signals that regulate protein O-GlcNAcylation are themselves under circadian and/or metabolic regulation

Cellular signals	Enzymes that regulate protein O-GlcNAcylation	Under circadian control?	Under metabolic control?
ER stress	Xbp1s	(217)	(218)
TGF-β		(184, 185)	(219, 220, 221, 222)
IL-8		(223)	(224, 225)
Angiotensin II		(180, 181)	(226)
Apoptosis	FOXA2	-	(227)
	CASP3	(228)	(229)
Oxidative stress	FAS	-	(230, 231)
Maturation of oocyte	NAT10	-	-
Cancer or other metabolic diseases	RANBP2-CEBPα	(98)	-
	ROCK2	(232)	(233)
	BAP1	-	-
	CUL3-NRF2	(234)	-
	miRNA	(235)	(236)
	cirRNA	-	-
Cytokinesis	ChK1	(237, 238)	-
Depolarization	CaMKIV	-	-
Norepinephrine	PERK	(239)	(240, 241)
EGF		(182, 183)	-
Infection	RELA-OGG1	(242)	(243, 244)
	FOXO1	-	(245)
	TG2	-	-
	E6AP	-	(246)
Hypoxia	HIF1α	(247, 248, 249, 250, 251)	(252)

Abbreviations: BAP1, BRCA1 associated protein-1; CASP3, caspase-3; CEBPα, CCAAT/enhancer-binding protein α; ChK1, checkpoint kinase one; circRNA, circular RNA; CUL3, cullin three; E6AP, ubiquitin ligase E6AP; EGF, epidermal growth factor; FAS, fatty acid synthase; FOXA2, forkhead box A2; FOXO1, forkhead box protein O1; HIF1α, hypoxia inducible factor 1α; IL-8, interleukin-8; miRNA, microRNA; NAT10, N-acetyltransferase 10; NRF2, nuclear factor E2-related factor-2; OGG1, 8-oxoguanine DNA glycosylase one; PERK, Protein Kinase RNA-like ER kinase; RANBP2, RAN-binding protein two; RELA, nuclear factor-κB p65; ROCK2, Rho-associated coiled-coil forming protein kinase two; TG2, transglutaminase two; TGF-β, transforming growth factor β; Xbp1, spliced X-box binding protein one.

## Conclusion and future prospects

Over 3 decades of investigations have advanced our understanding of the functions of O-GlcNAcylation in various fundamental cellular processes and pathological development of many diseases (5, 9, 10, 11, 12). Many studies have shown that O-GlcNAcylation is regulated by nutrient availability and nutrient sensing pathways (Fig. 1). In addition, there is rapidly accumulating evidence indicating that many other cellular signals and molecular pathways modulate protein O-GlcNAcylation (Figs. 2 and 3), suggesting the role of O-GlcNAcylation as a generalized cellular status effector. In this review, we discuss the metabolic regulation of protein O-GlcNAcylation through HBP and O-GlcNAc processing enzymes, summarize the mechanisms by which circadian clocks shape daily O-GlcNAcylation rhythms, and highlight other cellular signals and associated molecular mechanisms that influence cellular O-GlcNAcylation level. Our review emphasizes the importance of maintaining proper daily O-GlcNAcylation rhythm by controlling mealtime. O-GlcNAcylation is tightly regulated by daily nutrient input and circadian clocks and is expected to provide time-of-day specific regulation on cellular processes to mediate clock and metabolic control of daily rhythmic physiology and behavior (8). This review also provides insights into potential targets for the development of therapeutics to alleviate disruption of O-GlcNAc homeostasis in metabolic diseases. As metabolic input is the primary regulatory factor for O-GlcNAcylation, we propose that O-GlcNAcylation could partially mediate the beneficial effects of time-restricted eating in patients with metabolic syndrome, which limits the eating time to under 10 h during the active period (146). Notably, many regulatory factors of O-GlcNAcylation described in this review, such as ROCK2 and BAP1, have been already used as therapeutic targets in patients (147, 148).

In this review, we largely focused on the cellular signals and pathways that regulate overall protein O-GlcNAcylation. Besides the gaps in knowledge we have already highlighted in the previous sections, there remain many questions regarding the regulation of O-GlcNAcylation that warrant future investigations. One interesting question is how OGT and OGA achieve substrate specificity (149, 150), given there are thousands of protein substrates for O-GlcNAcylation. So far, it has been uncovered that mechanisms underlying substrate specificity include phosphorylation (94, 115, 151), subcellular localization (89, 90, 92), dimerization (152, 153, 154), structural divergence (111, 155, 156, 157, 158, 159, 160, 161), and adaptor proteins (6, 162, 163, 164, 165, 166). Another interesting question is how do other PTMs on target proteins, such as phosphorylation (167), acetylation (168, 169, 170), and ubiquitination (171, 172, 173) interact with O-GlcNAcylation in individual proteins to regulate structure and function. Among these PTMs, phosphorylation is the most well-studied PTM that can facilitate or inhibit protein O-GlcNAcylation depending on the position of amino acid residues (167, 174, 175, 176). Future studies examining the interactions between O-GlcNAcylation and other PTMs in response to metabolic and cellular signals are critical to provide a comprehensive understanding of the regulation of biological processes *via* multiple PTMs.

Beyond the cellular level, there have been more and more studies that investigated the impact of environmental, physiological, and systemic changes on protein O-GlcNAcylation in whole animal models. Development (30), aging (37), maternal stress (34), exercise (40, 141, 143, 177), cold exposure (116), fear conditioning (178) and rapid eye movement (REM) sleep deprivation (179) are all shown to influence protein O-GlcNAcylation. However, our knowledge of the underlying mechanisms is rather limited. In mouse skeletal muscle, exercise-induced lactate and hypoxia can signal to HIF1α, which increases OGT expression and O-GlcNAcylation (141). In mouse brown adipose tissue, cold exposure can trigger OGT phosphorylation by PERK and promote OGT activity (116). Perhaps not surprisingly, the O-GlcNAcylation response appears to be tissue-specific. For example, exercise upregulates O-GlcNAcylation in skeletal muscle (141, 177), but downregulates it in the heart (143). Therefore, more studies are warranted to investigate the mechanisms of O-GlcNAcylation regulation by various physiological and environmental conditions in animal models.

Finally, although the circadian clock has been shown to regulate daily O-GlcNAcylation rhythm (18, 26), the molecular mediator(s) are just starting to be explored (Fig. 2). Many regulators of O-GlcNAcylation mentioned in this review are under clock control, including angiotensin II (180, 181), EGF (182, 183), and TGF-β (184, 185) (Table 2). Many of them could play important roles in regulating rhythmic O-GlcNAcylation to control daily biological rhythms. It is important to point out that the daily O-GlcNAcylation rhythm is likely to be different in diurnal *versus* nocturnal animals, given their distinct feeding windows. In the same species, O-GlcNAcylation rhythm and its response to nutrient availability may display organ specificity, due to distinct nutrient sensitivity and metabolic properties of individual organs. For example, the blood-brain barrier renders the brain tissue less sensitive to the fluctuation of nutrients in peripheries, while the heart is extremely metabolically active so that it might be highly impacted by daily feeding activity. It is also interesting to investigate the impact of metabolic diseases on daily O-GlcNAcylation rhythm. As metabolic diseases, or even high-fat diet, are known to disrupt daily rhythms in the transcriptome, sleep/wake cycle, and feeding/fasting cycle (186, 187, 188, 189, 190, 191), O-GlcNAcylation might mediate the disruptive effect of metabolic diseases on circadian physiology. Finally, to characterize the function of O-GlcNAcylation in circadian physiology, it is critical to identify the proteins that are rhythmically modified by O-GlcNAcylation using proteomic approaches. Comparative O-GlcNAcome is warranted to reveal tissue-specific and/or pathologically disrupted rhythm of O-GlcNAc proteins. Additionally, since phosphorylation (29, 98, 192, 193), acetylation (194, 195), and ubiquitination (196) has been shown to oscillate over a 24-h period at the proteome level, proteome-wide interaction between rhythmic O-GlcNAcylation and other PTMs will also shed light on the regulation of O-GlcNAcylation rhythm on individual proteins.

## Conflict of interest

The authors declare that they have no conflicts of interest with the contents of this article.
